# Evidence that Transcriptional Alterations in *Sarcoptes scabiei* Are under Tight Post-Transcriptional (microRNA) Control

**DOI:** 10.3390/ijms23179719

**Published:** 2022-08-26

**Authors:** Pasi K. Korhonen, Tao Wang, Neil D. Young, Gangi R. Samarawickrama, Deepani D. Fernando, Guangxu Ma, Robin B. Gasser, Katja Fischer

**Affiliations:** 1 Faculty of Veterinary and Agricultural Sciences, The University of Melbourne, Parkville, VIC 3010, Australia; 2 Infection and Inflammation Program, QIMR Berghofer Medical Research Institute, Brisbane, QLD 4006, Australia; 3 School of Veterinary Science, University of Queensland, Gatton, QLD 4343, Australia; 4 Zhejiang Provincial Key Laboratory of Preventive Veterinary Medicine, College of Animal Sciences, Zhejiang University, Hangzhou 310058, China

**Keywords:** *Sarcoptes scabiei*, development, transcription, mRNA, microRNA

## Abstract

Here, we explored transcriptomic differences among early egg (Ee), late egg (Le) and adult female (Af) stages of the scabies mite, *Sarcoptes scabiei*, using an integrative bioinformatic approach. We recorded a high, negative correlation between miRNAs and genes with decreased mRNA transcription between the developmental stages, indicating substantial post-transcriptional repression; we also showed a positive correlation between miRNAs and genes with increased mRNA transcription, suggesting indirect post-transcriptional regulation. The alterations in mRNA transcription between the egg and adult female stages of *S. scabiei* were inferred to be linked to metabolism (including carbohydrate and lipid degradation, amino acid and energy metabolism), environmental information processing (e.g., signal transduction and signalling molecules), genetic information processing (e.g., transcription and translation) and/or organismal systems. Taken together, these results provide insight into the transcription of this socioeconomically important parasitic mite, with a particular focus on the egg stage. This work encourages further, detailed laboratory studies of miRNA regulation across all developmental stages of *S. scabiei* and might assist in discovering new intervention targets in the egg stage of *S. scabiei*.

## 1. Introduction

*Drosophila melanogaster* (vinegar fly or fruit fly) is (arguably) the best and most widely studied model arthropod [1]. Extensive investigations of this model fly have made it possible to gain deep, global insights into its developmental and reproductive biology, physiology, biochemistry, neurobiology and genetics [2,3,4]. By comparison, little is known about these areas for most parasitic arthropods of humans and other animals. Nonetheless, there have been major advances in the genomics and transcriptomics of arthropods (*n* > 76 species), including parasitic flies, fleas, lice, ticks and mites [5,6], based on detailed investigations. Recently, we sequenced and annotated the 57 Mb genome of *S. scabiei* (scabies mite) [7], providing a solid foundation for an array of future biological systems studies of this important pathogen. *S. scabiei* is the causative agent of human scabies, which is a neglected tropical disease (NTD) with a major adverse impact in disadvantaged communities around the world [8], particularly when associated with secondary bacterial infections and clinical complications.

The transmission of scabies usually occurs via the transfer of gravid females of *S. scabiei* from person-to-person (skin-to-skin) contact. Female mites burrow into the skin and deposit eggs (0.10 to 0.15 mm in length), which hatch within 3–4 days and release larvae that migrate to the skin surface and burrow into the stratum corneum. Larvae and ensuing nymphs occur within moulting pouches in the skin or hair follicles, and are markedly smaller than adult mites. Females are 0.25 to 0.35 mm wide and 0.30 to 0.45 mm long, and males are slightly more than half that size. Following male–female mating, impregnated females leave their moulting pouches and migrate on the cutaneous surface to find suitable locations to make serpentine burrows in the skin, where egg clutches are laid throughout the rest of the mites’ life (1–2 months). The eggs develop, and the life cycle is completed, again, via larvae and nymphs to adults.

The developmental biology of *S. scabiei* has not been studied in detail at the molecular level. Although some studies have explored some RNAs and/or proteins of *S. scabiei* [7,9,10,11], no investigation has yet compared transcriptomic profiles among particular developmental stages of this species or explored regulatory molecular processes. For instance, while small RNAs, such as microRNAs (miRNAs) [12,13], play key roles in regulating genes via mRNA silencing or chromatin modification [14,15], limited information has been available for *S. scabiei* [16] in relation to this parasite’s biology. Here, we explored mRNA and miRNA transcription profiles and correlations at early and late time points in the life cycle of the mite by employing a transcriptomic–bioinformatic approach, with a perspective on future investigations of host–mite interactions and the identification of targets for new acaricides against scabies [17].

## 2. Results

### 2.1. RNA Data Sets for Egg and Adult Stages of S. scabiei

Two datasets (i.e., mRNA-mapped genes and mapped miRNA reads) were produced for the Ee, Le and Af stages of *S. scabiei*. The numbers of mRNA reads that mapped to genes in the Ee, Le and Af stages were 120,050,416, 120,760,430 and 133,564,963, respectively (Table S1). Small RNA libraries yielded > 454,006,667 million reads, with 54 miRNAs being identified (Table S2), most of which were recorded for the first time in *S. scabiei*. The numbers of miRNA reads that represented the stages Ee, Le and Af and mapped to the *S. scabiei* genome (ASM1459567v1) were 11,512,297, 21,829,090 and 8,606,536, respectively (Table S1). No host contamination was detected amongst the mRNA or miRNA reads (Table S1).

### 2.2. Transcription in the Three Distinct Developmental Stages

**Figure 1:**
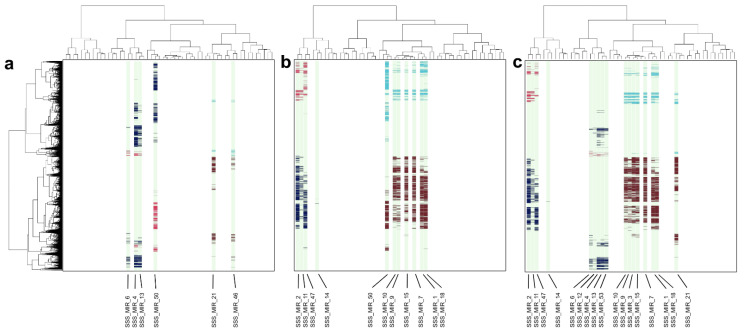
Correlation heatmap of the transcription levels (FC ≥ 1) of protein-coding genes (rows) and microRNAs (columns) across three developmental stages (i.e., early egg (Ee), late egg (Le) and adult female (Af)) of *Sarcoptes scabiei* upon pairwise comparison. The heatmaps (**a**) Ee vs. Le, (**b**) Le vs. Af and (**c**) Ee vs. Af display low (↓, dark blue and brown) and high (↑, light blue and red) mRNA transcription of genes associated with particular miRNAs (green lanes) (cf. Table 1 and Table 2). Vertical and horizontal dendrograms display similar transcription levels of protein-coding genes and miRNAs, respectively.

Hierarchical cluster analyses and multidimensional scaling resulted in a division of each transcriptomic (mRNA and miRNA) dataset into three groups representing the three developmental stages (Ee, Le and Af) of *S. scabiei* (Figure S1). The resultant clusters represented groups of genes whose functional enrichments were linked to individual miRNAs. The miRNAs associated with genes whose mRNA transcription was significantly lower or higher in a particular developmental stage (Ee, Le or Af) of *S. scabiei* relative to (i.e., vs.) another stage (upon pairwise comparison) are listed in Table 1 and Table 2 and are displayed in heatmaps (Figure 1a–c and Tables S3–S8).

Specifically, 2804 of 3669 (76.4%) genes with low mRNA transcription in Le vs. Ee were linked to four miRNAs (i.e., SSS_MIRs_4, 6, 13 and 50; Figure 1a and Table 1). These same four miRNAs were also linked to 1075 of 1326 (81.1%) genes with high mRNA transcription in Le vs. Ee (Figure 1a and Table 2). Similarly, 2425 of 3045 (79.6%) genes with low mRNA transcription in Af vs. Le associated with four distinct miRNAs (i.e., SSS_MIRs_2, 11, 14 and 47; Figure 1b and Table 1). These four miRNAs were linked to 781 of 2289 (34.1%) genes with high mRNA transcription in Af vs. Le (Figure 1b and Table 2). Finally, 3104 of 3778 (82.2%) genes with low mRNA transcription in Af vs. Ee associated with nine distinct miRNAs (SSS_MIR_2, 4, 6, 11, 12, 13, 14, 47 and 53; Figure 1c and Table 1); these nine miRNAs were linked to 580 of 737 (78.7%) genes with high mRNA transcription in Af vs. Ee (Figure 1c and Table 2). 

Similarly, 179 of 1326 (13.5%) genes with low mRNA transcription in Ee vs. Le were related to two miRNAs (SSS_MIRs_21 and 46; Figure 1a and Table 1). These two miRNAs were associated with 827 of 3669 (22.5%) genes with high mRNA transcription in Ee vs. Le (Figure 1a and Table 2). Moreover, 1840 of 2289 (80.3%) genes with low mRNA transcription in Le vs. Af were associated with seven different miRNAs (SSS_MIRs_1, 7, 9, 10, 15, 18 and 50; Figure 1b and Table 1); these seven miRNAs were linked to 2890 of 3045 (94.9%) genes with high mRNA transcription in Le vs. Af (Figure 1b and Table 2). Finally, 628 of 737 (85.2%) genes with low mRNA transcription in Ee vs. Af were associated with eight different miRNAs (SSS_MIRs_1, 3, 7, 9, 10, 15, 18 and 21; Figure 1c and Table 1); these eight miRNAs were linked to 2892 of 3778 (76.5%) genes with high mRNA transcription in Le vs. Af (Figure 1c and Table 2).

### 2.3. Biological Pathway/Process Analysis in Different Developmental Stages

KEGG pathway enrichment analysis was conducted to link biological functions to differentially transcribed genes and miRNAs (Tables S3–S10). This analysis revealed that the differentially transcribed genes (Tables S9 and S10) were associated with genetic and environmental information processing, organismal systems and/or metabolism.

Protein-coding genes that were highly transcribed in Ee vs. Le were linked to signal transduction; translation; protein folding, sorting and degradation; transcription; nucleotide replication and repair; the cell cycle; and glycan biosynthesis and metabolism (Tables S9 and S10). Protein-coding genes that were highly transcribed in Ee vs. Af were associated with development, regeneration and/or organismal systems (Tables S9 and S10). On the other hand, sets of miRNAs that were highly transcribed in Ee vs. Le (SSS_MIRs_21 and 46) and Ee vs. Af (SSS_MIRs_01, 03, 07, 09, 10, 15, 18 and 21) were linked to the downregulation of lipid, glycan, carbohydrate and/or amino acid metabolism (cf. Tables S3 and S5), and the same sets of highly transcribed miRNAs in Ee vs. Le (SSS_MIR_21 and 46) and Ee vs. Af (SSS_MIR_01, 03, 07, 09, 10, 15, 18 and 21) were linked to the upregulation of processes, predominantly signal transduction (Tables S4 and S6).

Protein-coding genes that were highly transcribed in Le (vs. either Ee or Af) were predominantly associated with signal transduction and organismal systems (Tables S9 and S10). Sets of miRNAs that were highly transcribed in Le vs. Ee (i.e., SSS_MIR_4, 6, 13 and 50) and Le vs. Af (i.e., SSS_MIR_01, 07, 09, 10, 15, 18 and 50) were linked to the downregulation of translation; protein folding, sorting and degradation; transcription (more generally); nucleotide replication and repair; the cell cycle; and glycan biosynthesis and metabolism (Tables S3 and S5). Interestingly, the same sets of miRNAs were highly transcribed in Le vs. Ee (i.e., SSS_MIR_4, 6, 13 and 50) and Le vs. Af (i.e., SSS_MIR_01, 07, 09, 10, 15, 18 and 50), and were linked to the upregulation of signal transduction and organismal systems (Supplementary Tables S4 and S6).

Protein-coding genes that were highly transcribed in Af vs. Ee were linked to lipid, glycan, carbohydrate, nucleotide and amino acid metabolism (cf. Tables S9 and S10). Conversely, such genes in Af vs. Le were associated with signal transduction; translation; protein folding, sorting and degradation; nucleotide replication and repair; the cell cycle; and glycan biosynthesis and metabolism (Tables S9 and S10). In addition, sets of miRNAs that were highly transcribed in Af vs. Ee (i.e., SSS_MIRs_02, 04, 06, 11, 12, 13, 14, 47 and 53) and Af vs. Le (i.e., SSS_MIRs_02, 11, 14 and 47) were linked to the downregulation of signal transduction and organismal systems (Tables S3 and S5). The same sets of miRNAs were highly transcribed in Af vs. Ee (i.e., SSS_MIR_02, 04, 06, 11, 12, 13, 14, 47 and 53) and Af vs. Le (i.e., SSS_MIR_02, 11, 14 and 47), and were associated with an upregulation of lipid, glycan, carbohydrate, energy and amino acid metabolism, as well as organismal systems and aging (Tables S4 and S6).

Finally, KEGG pathway enrichment analysis was conducted to link biological processes/pathways to genes that were predicted to be downregulated by microRNAs (Tables S11–S14). Using both seed pairing and site accessibility, 46 miRNAs associated with 416 genes and 589 miRNA–gene pairs were predicted (Table S12). These protein-encoding genes were linked to signal transduction and organismal systems in Ee vs. Le; signal transduction, organismal systems and DNA replication/repair in Le vs. Af; and signal transduction and organismal systems in Ee vs. Af (Tables S13 and S14). These findings were in accordance with those obtained from correlation-based analyses (Tables S3–S6).

## 3. Discussion

Here, we explored transcriptomic differences between eggs in the early and late phases of embryonation/development (i.e., Ee and Le) and the adult female (Af) stage of *S. scabies*. Pronounced transcriptional changes were seen, and plausible relationships were established between the transcription of protein-coding genes and miRNA transcription.

Initially, we predicted the miRNAs using the MirDeep2 program, which was applied to other invertebrates, such as fruit flies, nematodes and planarians, with a prediction accuracy of 98.6–99.9% [18]. Then, we employed two parameters (i.e., Pearson correlation and a *p*-value for permutations of samples) to correlate miRNAs with decreased or increased mRNA transcription. A high, negative correlation (r ≤ −0.7, *p*-value ≤ 0.001) between miRNAs and genes with decreased mRNA transcription between developmental stages indicated substantial post-transcriptional repression, whereas the positive correlation (r ≥ 0.7, *p*-value ≤ 0.001) between miRNAs and genes with increased mRNA transcription indicated marked post-transcriptional regulation. The use of the *p*-value for permutation across samples and replicates reduced the number of false positives in identifying confident associations. It is recognised that an indirect regulation by miRNA, such as the repression of some transcription factors (TFs), can lead to a down- or upregulation of protein-coding genes without miRNAs binding to them [19]. Here, through correlation analysis, we aimed to create a comprehensive ‘picture’ of miRNA-coupled associations, irrespective of whether the associations were direct or indirect. The method used for correlation analysis was similar to that employed by previous researchers [20,21,22]. Compared with the correlation approach used, the direct associations inferred using the Rna22 software inferred significantly fewer links between miRNAs and genes, as expected, which was likely also related to the fact that 3′-untranslated regions (UTRs) were known only for a subset of genes studied.

We proposed that subsets of miRNAs are post-transcriptional regulators [23,24,25] that play key roles in translational repression and mRNA degradation, which would explain the transcriptional patterns seen here in *S. scabiei.* Although we wanted to compare the miRNAs discovered here with those reported previously in a study that focused on immunobiology [16], this was not possible, as the sequence data for inferred miRNA from that study were not publicly available and the experimental conditions used in that study were distinct from those described here, preventing any direct comparison. Clearly, the present sets of miRNAs filled a significant knowledge gap and offer a useful new resource for future molecular investigations of *S. scabiei* and related mites.

The quantitative analyses carried out here revealed marked transcriptional differences between eggs and female adults of *S. scabiei*. Select functions were enriched in gene transcription and related miRNA regulation across these stages. In particular, the signalling and organismal systems enriched in the Ee and Le stages suggested the involvement of important developmental processes, similar to those involved in signal transduction and transcriptional regulation reported previously for *D. melanogaster* [13]. The transcription of molecules linked to metabolism in the Af stage reflected the mature mite established in the skin of the host organism. Although only 20 miRNAs with significant transcriptional differences among the three stages studied were identified, major changes in mRNA transcription were observed for thousands of protein-coding genes. In particular, pronounced differential transcription between the egg and adult female stages of *S. scabiei* reflected major alterations linked to this developmental transition. Overall, the findings suggested that six sets of miRNAs (cf. Table 1 and Table 2) were responsible for the up- or downregulation of transcription/translation, signal transduction and metabolism during this transition. However, this proposal requires experimental validation in the laboratory using methods such as a four-stage validation [26]. If validated, this could offer the prospect of discovering new intervention targets in egg stages. This focus is particularly relevant, given that current treatments against scabies do not kill the egg stage of *S. scabies* [8], which is central to the effective control of this disease.

Based on pathway enrichment analyses (cf. Tables S3 and S5), we proposed that signalling pathways (including Hippo, Notch, TGF-beta and Wnt) [27], glycan biosynthesis (N- and O- linked), lysosome trafficking, spliceosomes, translation factors, protein sorting/folding, RNA and ribosome biogenesis, the ubiquitin–proteasome system, threonine and cysteine peptidases, chromatin remodelling and histone processing could offer targets in the Ee stage *vis-à-vis* G-protein-coupled receptors, GPI-anchored proteins, serine/threonine phosphatases, ion channels and transporters, signal transduction molecules (e.g., MAPK) [28], cell adhesion molecules, cysteine peptidases and hydrolases in the Le stage. Interestingly, pathway enrichments for microRNA–gene pairs essentially matched those inferred following correlation-based analyses (not requiring knowledge of UTRs, which are usually missing from gene models inferred from short-read RNA data).

In conclusion, this investigation gave new insights into transcriptional differences between three developmental stages of *S. scabiei*. The present findings indicate that post-transcriptional regulation by key sets of miRNAs appear to play a critical role during the development of this mite. This developmental transition likely involves a range of biological changes that are reflected in signal transduction, genetic information processing and metabolism. Six key sets of miRNAs, proposed play critical regulatory functions in differentiation and development, were conspicuous and are suggested to also be involved in stress responses and/or environmental adaptation. These results encourage future laboratory-based studies of miRNA regulation in this important ectoparasite. The approach taken and the informatic workflow developed here should enable future studies of all life cycle stages of *S. scabiei*, host–mite interactions and, importantly, molecular responses of eggs and other stages of *S. scabiei* to new acaricidal treatments.

## 4. Materials and Methods

### 4.1. Production, Collection and Storage of S. scabiei Stages

*Sarcoptes scabiei* var. *suis* was produced on pigs (3 months of age), isolated and stored using well-established protocols [7,29]. Eggs at the early phase of embryonation (i.e., before the protolimbs were detectable using a light microscope (designated Ee)), eggs at the late phase of embryonation (when the protolimbs of the larvae within eggs were developed (designated Le)) and adult female mites (designated Af) were isolated from skin crusts. Five biological replicates of each Ee (*n* = 2500), Le (*n* = 1500) or Af (*n* = 500) were collected from skin crusts from experimentally infected pigs on different days.

### 4.2. RNA Extraction and Sequencing

Total RNA was isolated from the five replicate samples of each *S. scabiei* Ee, Le and Af (≤2 h after isolation from skin crusts) using the TRIzol™ reagent (cat. no. 15596026, Thermo Fischer Scientific Inc., Waltham, MA, USA) and treated with RNase-free TURBO DNase (Ambion^®^, cat no. AM1907, Thermo Fisher Scientific). Libraries for sequencing were constructed from mRNAs and small RNAs from each Ee, Le and Af using TruSeq Stranded Total RNA Library Prep Kit (Illumina Inc., San Diego, CA, USA) and TruSeq Small RNA Library Prep Kit (Illumina), respectively. In brief, mRNA was isolated using magnetic beads with Oligo (dT), cleaved into short fragments, transcribed into complementary DNA (cDNA) via reverse synthesis, connected with adaptors and amplified using PCR for mRNA-enriched libraries. For small RNA libraries, RNAs of 20–30 nucleotides (nt) in length were selected using electrophoresis, ligated to adaptors and amplified. All libraries were evaluated using the Agilent 2100 Bioanalyzer and ABI StepOnePlus real-time PCR system and then sequenced using the Illumina HiSeq™ 4000 platform.

### 4.3. Processing of mRNA and miRNA Data

The mRNA reads were assessed for quality using the program FASTQC v0.11.9 [30], and potential host contamination amongst mRNA reads was assessed using the program Centrifuge [31]; read data were then mapped to a predicted protein-coding gene set (NCBI accession identifier WVUK02000000) for the *S. scabiei* [7] genome (NCBI accession identifier WVUK00000000) using the program RSEM v1.3.3 [32] (parameters: --bowtie2 --no-bam-output --phred33-quals --paired-end) and counted. Multidimensional scaling [33] and hierarchical cluster analyses [34] were used to identify outliers. Strand-specific small RNA reads were also assessed for quality in the same manner, and miRNA reads (mature and precursor) were mapped to the respective reference genome [7], predicted and annotated using the program MirDeep2 v0.1.3 [18]. For the annotation of mature miRNA sequences, MirDeep2 was given miRNA data for the arthropods *Acyrthosiphon pisum, Aedes aegypti, Anopheles gambiae, Apis mellifera, Bombyx mori, Dinoponera quadriceps, Drosophila erecta, Drosophila melanogaster, Drosophila pseudoobscura, Drosophila sechellia, Drosophila simulans, Drosophila virilis, Drosophila yakuba, Parasteatoda tepidariorum, Plutella xylostella, Polistes canadensis* and *Tribolium castaneum* from miRbase (release 21) [35]. The numbers of miRNAs were established by mapping small RNA reads to predicted mature miRNAs using the program bowtie2 v7.5.0 [36] with the parameter setting ‘--very-sensitive-local’. Predicted miRNAs with mapped read hits were considered valid/genuine.

### 4.4. Differential Transcription, Correlating Differentially Transcribed Genes with miRNAs, miRNA-Target Relationships and KEGG Enrichment

Following log2-transformation, we assessed the correlation between the number of protein-coding genes whose mRNA transcription was either significantly lower or higher in a particular developmental stage of *S. scabiei* (relative to another) and the number of miRNAs using the Pearson correlation coefficient. The two-tailed *p*-value was calculated for each correlation value between a gene and a miRNA in the resultant Pearson correlation matrix by permuting samples 1000 times using R v4.1.2 [34]. Correlation values > 0.7 or <−0.7 with a *p*-value ≤ 0.001 were considered significant; correlation values outside of these ranges were set to zero. Differential gene and miRNA transcription between developmental stages of *S. scabiei* (relative to another) was established using the program EdgeR v3.36.0 [33] and limma v3.50.1 [37] employing a fold-change (FC) threshold of ≥1 and a *p*-value of <0.01. Then, differentially transcribed microRNAs (SSS_MIR codes) were correlated with genes whose transcription (mRNA) was recorded to be significantly lower or higher in a particular developmental stage (relative to another stage) of *S. scabiei*, i.e., in a pairwise manner between Ee, Le and Af. The correlation matrices for miRNA–gene pairs were displayed in heatmaps using R. In addition, miRNA-target predictions were explored using Rna22 software v2.0 [38]. KEGG enrichment analyses were conducted using established methods [39,40].

## Figures and Tables

**Table 1:** MicroRNAs (SSS_MIR codes; cf. Table S2) linked to genes whose transcription (mRNA) was significantly decreased (↓) in a particular developmental stage (relative to another stage) of *Sarcoptes scabiei* (cf. colour codes in Figure 1). Stages: Ee—early egg, Le—late egg (Le) and Af—adult female. Enriched biological pathways or processes that were inferred to be linked to reduced transcription are listed.

Description	↓ Le (Ee)	↓ Af (Le)	↓ Af (Ee)
No. of genes	3669	3045	3778
No. of genes linked to particular miRNAs	1403 (SSS_MIR_50) 978 (SSS_MIR_13) 269 (SSS_MIR_06) 1614 (SSS_MIR_04)	1338 (SSS_MIR_47) 8 (SSS_MIR_14) 555 (SSS_MIR_11) 2091 (SSS_MIR_02)	445 (SSS_MIR_53) 1108 (SSS_MIR_47) 7 (SSS_MIR_14) 667 (SSS_MIR_13) 110 (SSS_MIR_12) 557 (SSS_MIR_11) 265 (SSS_MIR_06) 726 (SSS_MIR_04) 2033 (SSS_MIR_02)
Total number of genes linked to respective miRNAs	2804	2425	3104
***Biological pathways or processes***			
Signal transduction	-	Ras, calcium, cAMP, PI3k-Akt, Rap1, MAPK	MAPK, cAMP, ErbB, PI3K-Akt, Rap1
Metabolism	-	-	-
Transcription/translation	Ribosome, RNA transport, DNA replication and repair, protein folding, sorting and degradation, spliceosome	-	-
Organismal systems	-	Nervous, circulatory, endocrine, digestive, sensory, leukocyte transendothelial migration	Nervous, circulatory, endocrine, digestive, sensory, leucocyte, transendothelial migration, development and regeneration
**Description**	**↓** **Ee (Le)**	**↓** **Le (Af)**	**↓** **Ee (Af)**
No. of genes	1326	2289	737
No. of genes linked to particular miRNAs (SSS code)	132 (SSS_MIR_46) 123 (SSS_MIR_21)	1601 (SSS_MIR_50) 566 (SSS_MIR_18) 306 (SSS_MIR_15) 292 (SSS_MIR_10) 314 (SSS_MIR_09) 368 (SSS_MIR_07) 568 (SSS_MIR_01)	276 (SSS_MIR_21) 382 (SSS_MIR_18) 370 (SSS_MIR_15) 345 (SSS_MIR_10) 347 (SSS_MIR_09) 395 (SSS_MIR_07) 362 (SSS_MIR_03) 432 (SSS_MIR_01)
Total no. of genes linked to respective miRNAs	179	1840	628
***Biological pathways or processes***			
Signal transduction	-	mTOR	PPAR
Metabolism	Lipid, carbohydrate	-	Lipid, carbohydrate, amino acid
Transcription/translation	-	Base excision repair, ribosome biogenesis, protein folding, sorting	-

**Table 2:** MicroRNAs (SSS_MIR codes; cf. Table S2) linked to genes whose transcription (mRNA) was significantly increased (↑) in a particular developmental stage (relative to another stage) of *Sarcoptes scabiei* (cf. colour codes in Figure 1). Stages: Ee:—early egg, Le—late egg (Le) and Af—adult female. Enriched biological pathways or processes that were inferred to be linked to increased transcription are listed.

Description	↑ Le (Ee)	↑ Af (Le)	↑ Af (Ee)
No. of genes	1326	2289	737
No. of genes linked to particular miRNAs	910 (SSS_MIR_50) 78 (SSS_MIR_13) 77 (SSS_MIR_06) 140 (SSS_MIR_04)	494 (SSS_MIR_47) 1 (SSS_MIR_14) 142 (SSS_MIR_11) 468 (SSS_MIR_02)	24 (SSS_MIR_53) 182 (SSS_MIR_47) 1 (SSS_MIR_14) 47 (SSS_MIR_13) 16 (SSS_MIR_12) 146 (SSS_MIR_11) 62 (SSS_MIR_06) 56 (SSS_MIR_04) 434 (SSS_MIR_02)
Total number of genes linked to respective miRNAs	1075	781	580
***Biological pathways or processes***			
Signal transduction	Calcium, cAMP, MAPK	-	-
Metabolism	-	Lipid, carbohydrate, amino acid, cofactors and vitamins	Lipid, carbohydrate, amino acid, energy, nucleotide
Transcription/translation	-	-	-
Organismal systems	-	-	Digestive, aging, antigen processing and presentation
**Description**	**↑** **Ee (Le)**	**↑** **Le (Af)**	**↑** **Ee (Af)**
No. of upregulated genes	3669	3045	3778
No. of genes linked to particular miRNAs (SSS code)	262 (SSS_MIR_46) 778 (SSS_MIR_21)	1219 (SSS_MIR_50) 1965 (SSS_MIR_18) 1733 (SSS_MIR_15) 1546 (SSS_MIR_10) 1275 (SSS_MIR_09) 1903 (SSS_MIR_07) 1887 (SSS_MIR_01)	1379 (SSS_MIR_21) 1812 (SSS_MIR_18) 1829 (SSS_MIR_15) 1611 (SSS_MIR_10) 1284 (SSS_MIR_09) 1936 (SSS_MIR_07) 1846 (SSS_MIR_03) 1813 (SSS_MIR_01)
Total no. of genes linked to respective miRNAs	827	2890	2892
***Biological pathways or processes***			
Signal transduction	Hippo, Notch, TGF-beta, Wnt, MAPK	Hippo, Ras, Apelin, cAMP, Calcium, PI3K-Atk Rap1, MAPK	Hedgehog, Hippo, cAMP, Ras, TGF-beta, PI3K-Akt, Calcium, Rap1, MAPK, Wnt
Metabolism	-	-	-
Transcription/translation	-	-	-
Organismal systems	Nervous, circulatory, endocrine, sensory, digestive	Nervous, circulatory, endocrine, sensory, digestive, Th1 and Th2 cell differentiation	Development and regeneration, nervous, endocrine, immune system

## Data Availability

All data from this study are available within this article.

## Supplementary Materials

The following supporting information can be downloaded from https://www.mdpi.com/article/10.3390/ijms23179719/s1, and R scripts and the miRNA GFF file can be downloaded from https://doi.org/10.26188/20436474.
