# Supplementary material for: Evidence that Transcriptional Alterations in Sarcoptes scabiei Are under Tight Post-Transcriptional (microRNA) Control
Source: Int J Mol Sci. 2022 Aug 26;23(17):9719. doi: 10.3390/ijms23179719 (PMC9456212; doi:10.3390/ijms23179719)
Supplement: Supplementary file 1 [file ijms-23-09719-s001.zip › ijms-1803442-supplementary/Supplementary files/Figure S1.pdf]

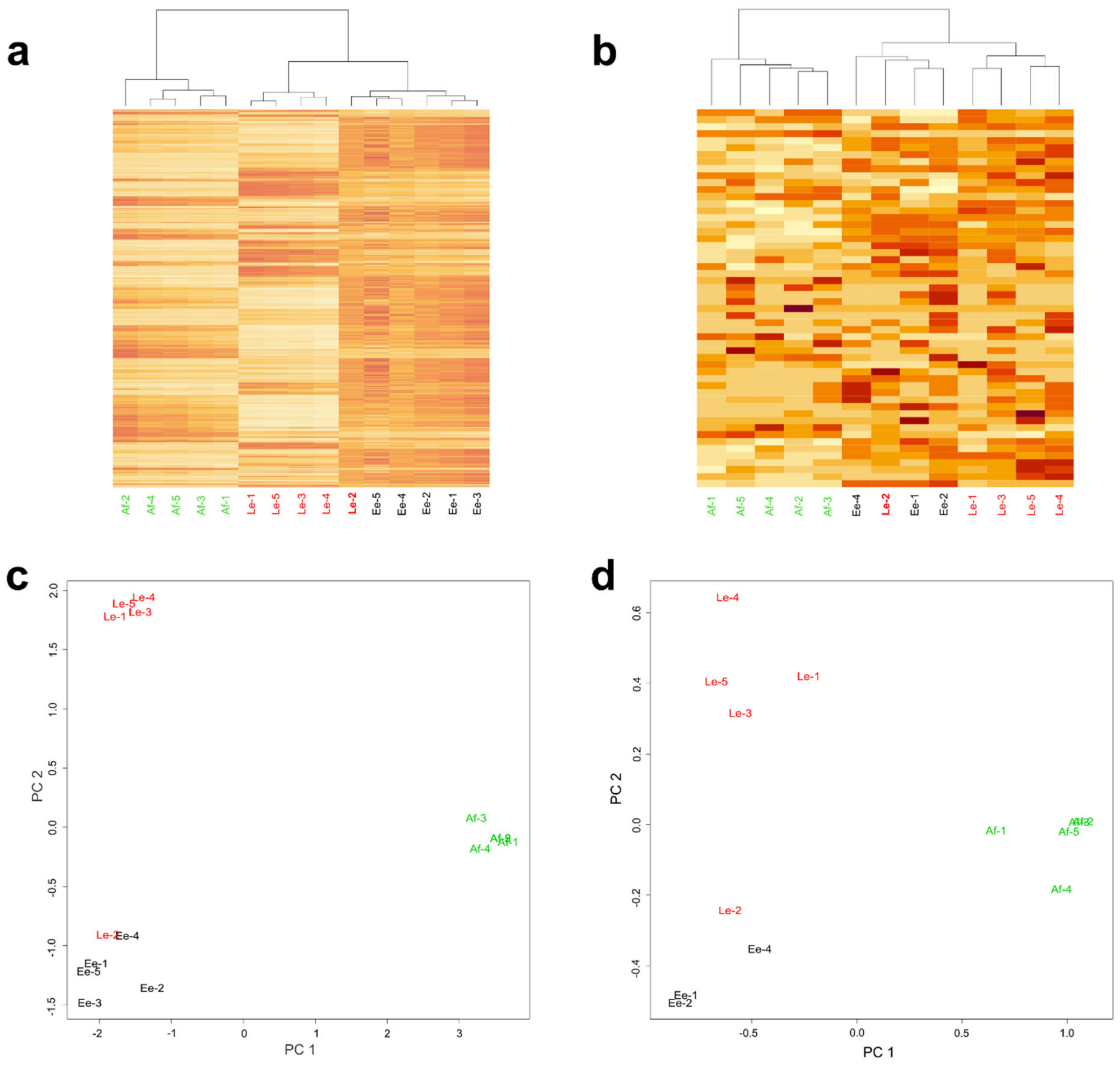

Figure S1. Hierarchical clustering of transcription profiles for protein-coding genes (**a**) and microRNAs (**b**) for three developmental stages of *Sarcoptes scabiei* – early egg [Ee; black], late egg [Le; red] and adult female [Af; green]). Multidimensional scaling plots of these genes (**c**) and microRNAs (**d**) clustered the samples and replicates into the same groups. The outlier (sample Le-2), visible in both representations, was removed from analyses in this study.
